# Local adaptation in the transgenerational response to copper pollution in the bryozoan *Bugula neritina*

**DOI:** 10.1002/ece3.9524

**Published:** 2022-11-15

**Authors:** Isabelle P. Neylan, Andrew Sih, John J. Stachowicz

**Affiliations:** ^1^ Department of Evolution & Ecology, Center for Population Biology UC Davis Davis CA USA; ^2^ Bodega Marine Laboratory UC Davis Davis CA USA; ^3^ Department of Environmental Science & Policy UC Davis Davis CA USA

**Keywords:** bryozoan, *Bugula neritina*, copper, local adaptation, transgenerational plasticity

## Abstract

Transgenerational plasticity (TGP)—when a parent or previous generation's environmental experience affects offspring phenotype without involving a genetic change—can be an important mechanism allowing for rapid adaptation. However, despite increasing numbers of empirical examples of TGP, there appears to be considerable variation in its strength and direction, yet limited understanding of what causes this variation. We compared patterns of TGP in response to stress across two populations with high versus low historical levels of stress exposure. Specifically, we expected that exposure to acute stress in the population experiencing historically high levels of stress would result in adaptive TGP or alternatively fixed tolerance (no parental effect), whereas the population with low levels of historical exposure would result in negative parental carryover effects. Using a common sessile marine invertebrate, *Bugula neritina*, and a split brood design, we exposed parents from both populations to copper or control treatments in the laboratory and then had them brood copper‐naïve larvae. We then exposed half of each larval brood to copper and half to control conditions before allowing them to grow to maturity in the field. Maternal copper exposure had a strong negative carryover effect on adult offspring growth and survival in the population without historical exposure, especially when larvae themselves were exposed to copper. We found little to no maternal or offspring treatment effect on adult growth and survival in the population with a history of copper exposure. However, parents from this population produced larger larvae on average and were able to increase the size of their larvae in response to copper exposure, providing a potential mechanism for maintaining fitness and suggesting TGP through maternal provisioning. These results indicate that the ability to adjust offspring phenotype via TGP may be a locally adapted trait and potentially influenced by past patterns of exposure.

## Graphical abstract

Transgenerational plasticity (TGP) may be an adaptive strategy to cope with rapid environmental changes or cause negative carryover effects that reduce offspring fitness. While TGP occurs across a broad variety of taxa, we have limited understanding of the conditions under which TGP is adaptive, in part because this requires significant knowledge of the environmental history of a population, which is rarely well‐characterized. We tested a priori predictions about the strength and nature of TGP among two populations of the sessile marine invertebrate *Bugula neritina* with different known histories of stress exposure. We found two distinct patterns of transgenerational responses to this stress indicating that the ability to adjust offspring phenotype via TGP may be a locally adapted trait and potentially influenced by past patterns of exposure.

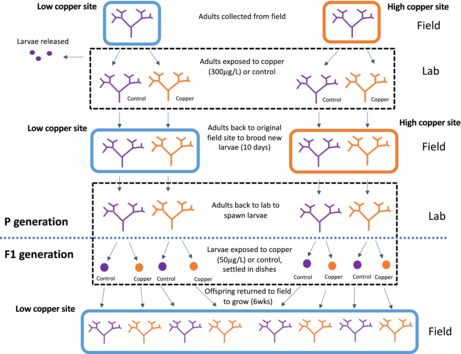

## 1 INTRODUCTION

Transgenerational plasticity (TGP) occurs when a change in offspring phenotype or reaction norm is caused by an environment experienced by the parent (or previous generations) without involving a genetic change (Holeski et al., 2012; Salinas et al., 2013). This form of non‐genetic inheritance is taxonomically widespread (Jablonka & Raz, 2009; Salinas et al., 2013) and is, in theory, as universally applicable as genetic inheritance (Day & Bonduriansky, 2011). Changes can persist for several generations, act on ecologically relevant traits, and can have population‐level consequences (Bell & Hellmann, 2019; Bossdorf et al., 2008; Donelan et al., 2020). However, despite increasing numbers of empirical examples of TGP, there are few tests exploring what conditions make it an adaptive strategy and whether theoretical predictions surrounding its evolution are supported in nature. In particular, the strength of adaptive TGP is quite variable across species and among populations within a species (Uller et al., 2013; Yin et al., 2019) suggesting that the context of the system must be taken into account when studying these patterns. Yet few studies of TGP consider more than a single species or population, limiting our ability to infer drivers of this variation beyond theoretical predictions.

A population's evolutionary history with a given environmental factor is one such context that should influence whether this form of plasticity is adaptive and dictate what transgenerational patterns emerge (Colicchio & Herman, 2020; Walsh et al., 2016). When the environmental factor is a stressor (e.g., pollutants), there are two main types of transgenerational outcomes. A stressful environmental factor will likely reduce parental condition, which can then carry over to the next generation with parents creating lower‐quality offspring. The stressor creates a negative parental effect in the form of a condition‐dependent constraint (negative carryover effects). Alternatively, the same factor could also serve as a cue that allows parents to prime their offspring to better cope with that stressor. In this case, TGP increases offspring performance as parents compensate for the stressful conditions the next generation is likely to face (via maternal priming or adaptive matching) (Sobral et al., 2021; Uller et al., 2013).

**FIGURE 1:**
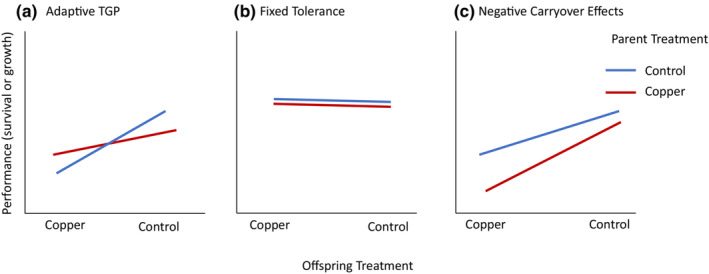
Graphs illustrating hypothetical responses to stress with offspring performance (for example survival or growth) on the *y*‐axis, offspring or F1 treatment on the *x*‐axis (larvae exposed to either copper or control), and the color of the lines signifying the parent treatment (control in blue and copper in red). (a) A possible adaptive transgenerational response with parental experience with copper conferring a benefit to offspring also exposed to copper. (b) No transgenerational effects, all offspring perform comparably regardless of parental or F1 larval treatment. (c) Another possible transgenerational response, a negative carryover effect, with larvae exposed to copper who also had a parent exposed to copper performing worst.

An open question lies in predicting which of these two outcomes will dominate under different stress intensities and durations. For example, if a population lacks a history of consistent stress exposure, there may not have been selection favoring the ability to prime offspring. These parents would likely also lack the ability to cope with the stressor themselves, which will make it more likely that they will produce lower‐quality offspring in response to novel stresses; thus, negative carryover effects may dominate in naïve populations. In contrast, if there is a history of exposure, parents may have evolved adaptations to reduce stress and thus reduce the transgenerational negative carry over effects to offspring. Furthermore, parents in exposed populations may have evolved the ability to prime their offspring for success under exposure conditions. Alternatively, if the adaptation in exposed populations is sufficiently strong that the stressor no longer has much of an impact on offspring fitness, then there may be no transgenerational plasticity or parental effects at all (Figure 1).

We assessed the transgenerational response of two neighboring populations of the Bryozoan *Bugula neritina* that have documented differences in their stress exposure histories. *Bugula neritina* is a regularly branching, colonial marine invertebrate that is a cosmopolitan and highly invasive member of the fouling community (sessile marine organisms that commonly grow on human‐made structures such as docks, pilings, and boat hulls). Each adult colony is composed of many clonal units (zooids). Colonies grow through asexual creation of new zoids while new colonies are formed through sexual reproduction (Keough, 1989). After fertilization through broadcast spawning of male gametes, females brood larvae until they are ready to be released (Marshall, 2008; Marshall et al., 2006). Larvae are lecithotrophic, or non‐feeding, and swim for a short period of time (typically between 15 min to 3 h) before settling onto the substrate and beginning metamorphosis into an ancestrula that will give rise to the adult colony via asexual budding (Lynch, 1947). *Bugula neritina* is well‐suited to testing the strength and direction of transgenerational plasticity as it has a short dispersal distance, grows quickly, has a short generation time, reproduces rapidly, and readily releases larvae that settle and undergo metamorphosis in the lab.

A stressor frequently encountered by *B. neritina* is copper toxicity. It has been common practice to use anti‐fouling paints on boat hulls and other surfaces to prevent the unwanted growth of pest‐fouling species such as *B. neritina* (Schiff et al., 2004; Srinivasan & Swain, 2007). These paints frequently contain heavy metals (including copper) that leach into the water causing negative fitness effects on marine invertebrates (Brooks & Waldock, 2009; Wisely, 1963; Wisely & Blick, 1967). These coated hulls can passively leach copper for up to 6 months after initial application with boat owners advised to clean their hulls and reapply at least once a year or as often as every month (Valkirs et al., 2003). Depending on the leach rate of the paint and how close an organism has settled, ambient copper concentrations can reach levels which have been shown to negatively affect multiple Bryozoan species (Miller, 1946; Piola & Johnston, 2006b). *Bugula neritina* is considered more copper tolerant than many other fouling species making ambient copper levels sublethal, but still deleterious. Early‐life history exposure to copper has significant effects on *Bugula* larval performance, subsequent growth, and survival in the adult colonies (Miller, 1946; Piola & Johnston, 2006a; Wisely, 1963).

Beyond the within‐generational effects of copper exposure, previous work with *B. neritina* has shown that there may be adaptive maternal effects for mothers exposed to copper that had offspring that also experienced elevated copper levels (Marshall, 2008). One possible explanation is the strong connection between larval size (dictated by maternal provisioning) and subsequent fitness (Marshall et al., 2006; Marshall & Keough, 2003). The ability of the parent to provision their offspring appropriately may be vital to their survival through the vulnerable larval stage and their ability to reach a location suitable for metamorphosis and adult survival. Parental exposure to stress, including copper, affects larval size in the next generation (Marshall, 2008). Importantly, *B. neritina* populations can be locally adapted to a given copper regime (Piola & Johnston, 2006a), and in a few cases in other taxa, the expression of TGP has been shown to vary across populations with differing environmental and evolutionary histories (Münzbergová & Hadincová, 2017; Walsh et al., 2016).

To assess variation in TGP among populations, we ran a fully crossed factorial, split‐brood experiment with a copper‐seawater solution and control seawater treatments representing a heavily copper‐polluted harbor and a relatively unpolluted harbor, respectively. This design allowed us to test whether two populations responded differently when assessed simultaneously using identical methods. These exposures were carried out at the parents' (F0) adult reproductively mature stage and at the offspring's (F1) larval stage. The adult *B. neritina* (F0) colonies were collected from the wild from two sites located within a few kilometers of each other in San Diego Bay, CA. We chose this pair of sites as one site is known to have historically higher levels of ambient copper while the other site has lower levels of copper (Blake et al., 2004; Schiff et al., 2007). This historical data of stress exposure provided a rationale for expecting why there might be variation among populations and allowed us to evaluate whether such variation was consistent with our hypotheses about how historical stress levels should affect TGP. Specifically, we expected that in a population with a higher exposure to copper, there would be a higher likelihood of adaptive TGP than in the copper‐naïve population (Figure 1a). However, a plausible alternative hypothesis is that if the exposure was constant and unvarying, selection might favor fixed tolerance to high copper and no TGP (Figure 1b). At the lower copper site, we expected no evidence of adaptive TGP and instead, predicted negative parental carryover effects (Figure 1c). We also predicted that maternal provisioning through manipulation of larval size may be one possible mechanism explaining any transgenerational patterns we find.

## 2 METHODS AND MATERIALS

### 2.1 Study sites

We collected adult colonies of *B. neritina* from two sites around Shelter Island in San Diego Bay, California, USA. The first site was within the Shelter Island Marina (32°43′05.9′′N 117°13′29.1′′W), a site known to have historically high levels of ambient copper between 3–14 μg/L on average (Blake et al., 2004; Schiff et al., 2007) and considered a copper pollution “hotspot” since at least the late 1970 s (Phillips, 1988; Stevens, 1988; Van der Weele, 1996). The abundance of large yachts and boats in combination with a long water residence time is thought to contribute to these elevated copper levels with copper concentrations increasing with distance from the mouth of the marina (California Regional Water Quality Control Board San Diego Region 2005). Importantly, these elevated levels of copper have been impacting this population of *B. neritina* for many generations. This site will be referred to as the Marina site hereafter. The second site was a public boat launch on the opposite side of Shelter Island with documented copper levels averaging 0–4 μg/L (Blake et al., 2004). Unlike the first site, this Boat Ramp site (32°42′54.8′′N 117°13′23.7′′W) lacks long‐term anchored boats and has greater water exchange with the open sea, flushing out any potential pollutants. Importantly, these two sites are <0.5 km apart by land and are similar in several important environmental metrics (e.g., salinity and temperature). However, a larva traveling through the water would have to cover approximately 3 km to reach one site from the other due to the local geography. While there is a possibility for gene flow between the two populations, given the short larval duration of this species and the lack of strong currents between the two sites, conditions support the presence of population differentiation.

We collected seawater samples from each site during the experimental deployment at high and low tide and analyzed them for copper concentration using mass spectrometry at UC Davis Interdisciplinary Center for Plasma Mass Spectrometry. These tests revealed higher copper levels at the Marina site (7.36 ± 0.76 μg/L [mean ± SE]) than at the Boat Ramp site (3.85 ± 1.92 μg/L [mean ± SE]) as expected. Copper concentrations can fluctuate dramatically, in particular at the Marina site that houses a large number of boats, with localized pulses that can be much higher than these ambient levels (Schiff et al., 2004; Stauber et al., 2000). In laboratory studies, acute copper exposure at levels as high as 25–100 μg/L can have sublethal but detrimental effects on *B. neritina* performance, particularly when exposed in early life as larvae, with adult colonies able to tolerate copper levels as high as 500 μg/L (Marshall, 2008; Miller, 1946; Piola & Johnston, 2006a). Chronic exposure to lower copper levels, however, has also been shown to impede growth and reduce survival (Piola & Johnston, 2006a).

### 2.2 Organism collection and maintenance

We collected mature colonies along approximately 100 m of dock at each site and brought them to the San Diego State University's Coastal and Marine Institute Laboratory (CMIL) for spawning in September 2019. We maintained adults in a cooler during transport before transferring them to a flow‐through seawater table at the lab.

### 2.3 Equipment preparation

We washed all equipment in 5% nitric acid for at least 24 h and triple rinsed in Milli‐Q water prior to use to remove any copper that might be present. Copper II sulfate pentahydrate (CuSO_4_) was our reference toxicant. Artificial seawater (Instant Ocean + Milli‐Q filtered water) was used to make all stock solutions to avoid contamination from organic matter found in natural seawater that can cause complexation of copper and vary its concentration and bioavailability (Ng & Keough, 2003). A 1000 μg/L stock solution was prepared and kept refrigerated at 4°C when not in use to prevent reduction (Marshall, 2008; Piola & Johnston, 2006b). We then diluted this stock solution to the desired concentration on the day of each trial using filtered seawater. We pre‐soaked any experimental containers in their respective solutions overnight (~12 h), then replaced the solution prior to introducing animals to minimize the potential chelation of the copper to the surface of the containers, which would reduce its concentration in the solution (Marshall, 2008; Piola & Johnston, 2006b).

### 2.4 Experimental design

**FIGURE 2:**
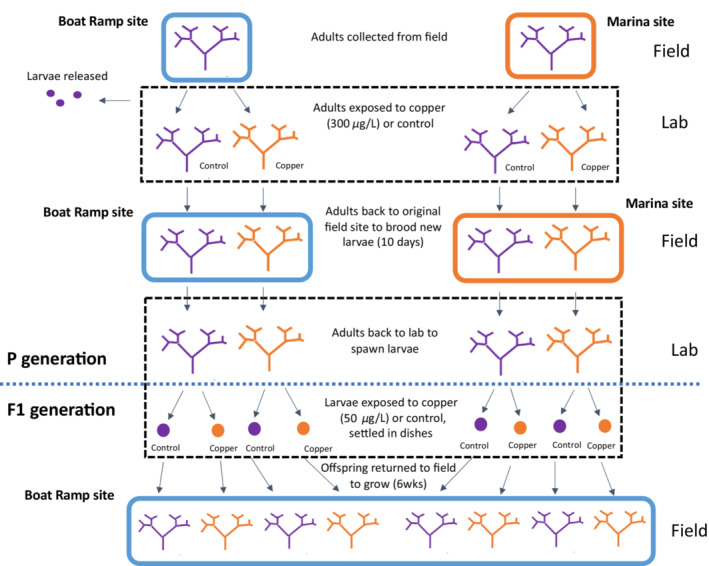
A schematic illustrating the experimental design. Adult colonies were collected and brought back to the lab from two different field sites (Boat Ramp site, blue; Marina site, orange), induced to release any currently brooded larvae, and exposed to either a copper solution (300 μg/L) or control (filtered seawater). The colonies were deployed back to their respective field sites to brood another batch of larvae. They were then brought to the lab again and induced to spawn. The resulting larvae were split between petri dishes containing copper solution (50 μg/L) or control (filtered seawater) and allowed to settle and metamorphose before being transferred back to the Boat Ramp field site where survival and growth were tracked weekly for 6 weeks.

We ran a fully crossed factorial, split‐brood experiment with a copper‐seawater solution and control seawater as treatments representing a heavily copper‐polluted harbor and a relatively unpolluted harbor. These exposures were carried out at the parents' (F0) adult, reproductively mature stage and at the offsprings' (F1) larval stage (Figure 2). Details of exposure protocols at each stage are described below.

### 2.5 Maternal (F0) copper exposure

We first needed to remove all larvae from the adult colonies to ensure that we could separate the effects of direct embryonic or larval exposure to copper from the effects of parental exposure. We collected adult colonies (60 from each site) and kept them in the dark in flowing seawater for 24 h before spawning (Marshall, 2008). The following morning, we exposed the colonies to bright fluorescent light, which resulted in larval release. Colony collection and spawning occurred in the morning to standardize timing across trials and to maximize spawning success (Lynch, 1947). Colonies generally began to spawn within 10 min of exposure to light with the majority of larvae released after around 30 min.

After an hour of larval release, we held the larvae‐free adults in the copper solution (300 μg/L) or filtered seawater (control) for an additional 6 h (Marshall, 2008; Ng & Keough, 2003) in 570 ml glass jars filled with 200 ml of control or copper solution. This concentration would represent a pulse event on the upper end of the spectrum of what a colony would experience in the field (Teasdale et al., 2003). We chose this concentration to maximize the potential for detectable effects in subsequent generations and to allow for comparisons with previous *B. neritina* transgenerational work by Marshall (2008) who also used this concentration on parents. After copper exposure, we returned the adult colonies to the dock at their origin site for 10 days to allow for development and brooding of new offspring (Marshall, 2008). Although brooding larvae were potentially exposed to copper during these 10 days, the ambient copper concentrations at both field sites were substantially lower than the experimental levels experienced by both adults and larvae in the laboratory, and it was necessary to return the adult colonies to their original sites to ensure that both parents for each F1 cross came from within the same population. We suspended the colonies upside‐down about a meter below the water by inserting the base of each colony between strands of triple‐stranded rope strung across PVC pipes. There were no lethal effects of the copper exposure or dock deployment on maternal colonies and neither treatment affected later spawning success.

### 2.6 Larval (F1) copper exposure

After the 10‐day deployment in the field, we returned the adult colonies (60 per site, 30 in each treatment) to CMIL and kept them in the dark in flow‐through seawater as before. After 24 h, we exposed the adults to bright light in separate vessels and after 30 min collected the larvae using a syringe. From each adult colony, we collected 24 larvae; we placed 12 larvae into a petri dish containing a 50 ml copper solution (50 μg/L) and another 12 larvae placed in a dish containing 50 ml filtered seawater (control) for a split brood design (Figure 2). We chose this copper concentration as it represents a strong, but sublethal amount of toxicant that mirrors the concentrations used in other studies examining impacts of copper exposure on *B. neritina* larvae (Marshall, 2008; Piola & Johnston, 2006b) and would represent a high but plausible level observed in the field (Schiff et al., 2004; Stauber et al., 2000). Each parent (F0) × larval (F1) treatment had 30 petri dishes (each 100 mm diameter × 15 mm deep) across two populations for a total of 240 dishes and approximately 3500 larvae total. We roughened the inside surfaces of each dish with sandpaper and soaked them in seawater to create a biofilm for at least 24 h to provide a more suitable settling substrate (Marshall & Keough, 2003).

We left larvae undisturbed in the dark for 24 h to promote settling (Marshall et al., 2003) and counted the number of successfully metamorphosed individuals after 24 h and 48 h. By 24 h, the majority of larvae had settled and by 48 h the majority of those had metamorphosed successfully. We then standardized the number of settlers to 8–10 per dish to ensure consistent initial density across replicates and treatments. All successfully metamorphosed settlers were circled using a pencil so that they could be distinguished from new settlers that occurred after deployment (see below).

### 2.7 Field experiment (F1)

We deployed settled larvae in the field at the Boat Ramp site to keep copper exposure low as the colonies grew so as not to confound our interpretation of the effects of exposure during the larval phase with that during colony growth. We attached each petri dish to a plastic ~60–90 cm backing board using super glue and Velcro® with 40 dishes per board. We suspended each board face‐down in the water (to minimize sedimentation in the dishes) at about 1 m below the water line (Wendt, 1998).

We measured growth and survival of the F1 colonies weekly for 6 weeks by counting the number of bifurcations along the longest branch, which is an accurate proxy for total colony growth and mass (Keough & Chernoff, 1987) and fecundity (Marshall & Keough, 2003). To ensure accuracy and minimize the amount of time animals spent out of the water, we transported petri dishes detached from their boards to the lab and then maintained them in flow‐through seawater except when measuring that dish. In addition, we scraped each dish clean of any additional fouling organisms to help identify the focal individuals and standardize conditions across each dish as competition has been shown to affect growth and survival in *B. neritina* and can potentially confound maternal effects due to copper exposure (Marshall, 2008). After measurement, we returned the plates back to haphazardly selected positions on each board in the field to avoid any confounding effects of position.

### 2.8 Larval (F1) size measurements

We also measured larval size from 10 additional adult colonies from each population exposed to each treatment. We collected adult colonies from the same field locations and spawned them at the lab using the same protocols as described above. We used larvae collected during the first spawning (pre‐copper treatment) to measure a baseline size and then measured the size of larvae from these same mothers after copper exposure and a 10‐day field deployment as above. We measured length along the cilial groove and the widest perpendicular line to the groove as per the methods in Marshall et al. (2006) using Fiji software on photographs of individual larvae taken under a microscope at 35× magnification (Schindelin et al., 2012). We photographed and measured 15–20 larvae per colony both before and after exposure to copper (*n* = 150–200 measured larvae per time point per population).

### 2.9 Statistical analyses

The effect of copper on offspring survival after 6 weeks in the field was a binary response per individual (total *n* = 1924), and we analyzed these data using Generalized Linear Mixed Models assuming a binomial distribution (logit link function) with Kenward‐Roger approximation of degrees of freedom. Fixed factors included population (Marina or Boat Ramp site), parental treatment (copper or control), offspring treatment (copper or control), and the interactions between these factors including a three‐way interaction. We included parent identity and petri dish as nested random factors. We began by analyzing full models and then used stepwise deletion following the AIC criterion until we found the minimal adequate models to avoid overfitting. We were concerned that the large difference in survival between the two populations across all treatments may be driving the overall patterns in these models and overshadowing other possible patterns. Since we were interested in differences between populations in maternal and offspring effects and their possible interactions, GLMMs were also run for each population separately following the same stepwise model selection process described above. We assessed the significance of fixed effects by analysis of deviance, Type II Wald chi‐square tests using the ANOVA function from the car package (Fox & Weisberg, 2019).

We assessed the effect of copper on the growth of individual offspring after 6 weeks with linear mixed models using maximum likelihood (those that survived to 6 weeks, *n* = 934). We used the same procedure for model selection using the AIC criterion as described above. We assessed the significance of fixed effects by analysis of deviance, Type II Wald chi‐square tests using the ANOVA function as above.

We assessed variation in larval size from mothers pre‐ and post‐copper exposure using linear models given the Gaussian distribution of the data with area (calculated by multiplying the length and width measurements of each larvae) as the response variable, and origin population and before vs. after copper exposure as fixed factors. We further explored differences in larval size before and after parental exposure within each population using a pairwise comparisons with a Bonferroni correction (“contrasts”; package emmeans; Lenth [2022]). We performed all analyses in R version 3.5.1 using the lme4 package (Bates et al., 2015).

## 3 RESULTS

### 3.1 Survival of offspring after 6 weeks

**FIGURE 3:**
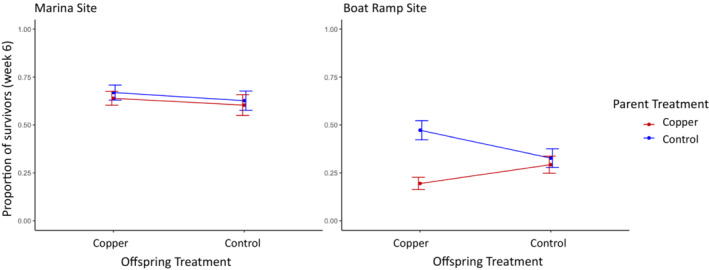
The proportion of surviving F1 offspring after 6 weeks in the field. Offspring treatment is represented along the *x*‐axis (copper or control), and parental treatment is represented by the line colors (copper in red, control in blue). The adult parent colonies were collected from two sites, one with historically higher copper levels (Marina site) and one with lower copper levels (Boat Ramp site). Error bars represent standard error.

We found a strong effect of population origin and maternal treatment on offspring survival indicating that both recent and historical exposure influenced fitness. Offspring from mothers collected at the Marina site had a higher proportion of survivors across treatments than those collected from the Boat Ramp site (GLMM, binomial; *p* < .001; Figure 3; Table 1). In addition, offspring from mothers that were not exposed to copper had higher survival. There was also a significant interaction between parental treatment and offspring treatment (GLMM, binomial; *p* = .036) and between parental treatment and population (GLMM, binomial; *p* = .023); we evaluate these interactions below in models run separately for each population. There were no effects of offspring treatment, offspring treatment by population interaction, or the three‐way interaction between offspring treatment, parental treatment, and population (Table 1).

**TABLE 1:** Parameter estimates from the binomial GLM examining the survival of F1 offspring after 6 weeks in the field across both Marina and Boat Ramp populations.

Fixed effects	Estimates (SE)	df	F (K‐R estimation)	Pr(<|z|)
Parent treatment	1.5029 (0.4322)	1	11.274	**<.001**
Offspring treatment	0.4696 (0.3399)	1	0.001	.167
Population	2.2013 (0.3766)	1	58.233	**<.0001**
Parent × Offspring	−1.1777 (0.5608)	1	2.001	**.0357**
Parent × Population	−1.2739 (0.5584)	1	2.965	**.0225**
Offspring × Population	−0.3761 (0.4649)	1	0.049	.4185
Parent × Offspring × Population	1.0987 (0.7203)	1	2.378	.1272
**Random effects**	**Variance**	**SD**		
Parent ID	0.7803	0.8834		
Dish ID × Parent ID	0.6031	0.7766		

*Note*: *p*‐Values with significant results (*p* < .05) shown in bold.

Separate binomial GLMMs for each population revealed treatment effects only in the Boat Ramp population. In the Marina population, larval survival was unaffected by either adult or larval exposure and the model with only random effects was the best fit to the data. In the Boat Ramp population, however, we found effects of parental treatment. This treatment effect in the Boat Ramp population (Table 2; Figure 3), and lack of such effects in the Marina population, caused the significant interactions in the combined population model. Parental exposure to copper decreased subsequent F1 survival in the Boat Ramp population (GLMM, binomial; *p* = .003), but offspring exposure (*p* = .539) did not and there was no interaction between parental and offspring treatment (*p* = .08).

**TABLE 2:** Parameter estimates from the binomial GLM examining the survival of F1 offspring after 6 weeks in the field originating from the Boat Ramp site.

Fixed effects	Estimates (SE)	df	F (K‐R estimation)	Pr(<|z|)
Parent treatment	1.4711 (0.4946)	1	0.6792	**.003**
Offspring treatment	0.2000 (0.3257)	1	0.1757	.539
Parent × Offspring	−0.9814 (0.5609)	1	0.0840	.080
**Random effects**	**Variance**	**SD**		
Parent ID	1.8422	1.357		
Dish ID × Parent ID	0.4409	0.664		

*Note*: *p*‐values with significant results (*p* < .05) shown in bold.

### 3.2 Growth of offspring after 6 weeks

**FIGURE 4:**
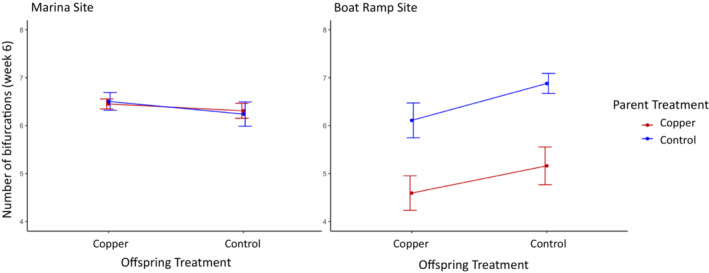
The growth of F1 offspring after 6 weeks in the field as measured by the number of bifurcations on the longest branch of each surviving colony. Offspring treatment is represented along the *x*‐axis (copper or control), and parental treatment is represented by the line colors (copper in red, control in blue). The adult parent colonies were collected from two sites, one with historically higher copper levels (Marina site) and one with lower copper levels (Boat Ramp site). Error bars represent standard error.

As with survival, the best model for growth of surviving offspring only included population, parental treatment, and their interaction. There was a significant effect of population on growth in F1 offspring after 6 weeks in the field (LMM; *p* < .05; Figure 4; Table 3). However, unlike with the survival data, this does not appear to be due to a large difference in mean growth (Marina site mean bifurcations across treatments = 6.34, Boat Ramp site mean across treatments = 6.21), but rather the increased variance in the Boat Ramp site driven by an effect of parental treatment on growth (LMM; *p* = .016; Table 3). Interestingly, there was no effect of offspring treatment on subsequent growth (LMM; *p* = .54). Overall, maternal exposure to copper decreased growth, but only in individuals from the Boat Ramp site.

**TABLE 3:** Parameter estimates from the LMM examining the growth of F1 offspring (measured in average number of bifurcations per colony) after 6 weeks in the field across both the Marina and Boat Ramp populations.

Fixed effects	Estimates (SE)	df	Pr(<|t|)	Pr(>Chisq)
Parent treatment	1.0922 (0.426)	1	**.0115**	**.0463**
Population	1.0962 (0.377)	1	**.0045**	**.0159**
Parent × Population	−0.9379 (0.5638)	1	.0992	.0962

*Note*: *p*‐values with significant results (*p* < .05) shown in bold.

### 3.3 Larval size in response to maternal copper exposure

**FIGURE 5:**
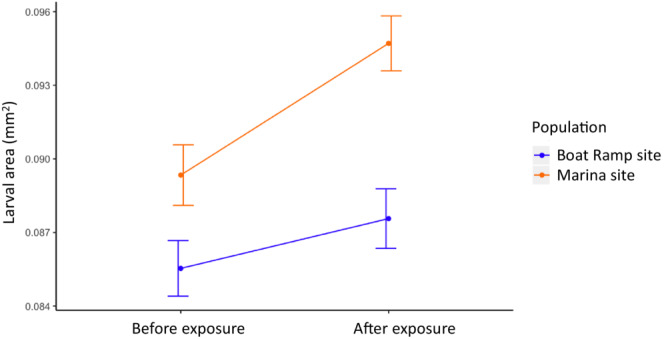
Larval size of F1 offspring from the same mothers before and after exposure to copper. Area calculated by multiplying the length of each larva measured along the cilial groove and the width of the widest part of the body perpendicular to the cillial groove to get larval area. The adult parent colonies were collected from two sites, one with historically higher copper levels (Marina site) and one with lower copper levels (Boat Ramp site) represented here with line colors (Boat Ramp site in blue, Marina site in orange). Error bars represent standard error.

Overall, the Marina site mothers produced 5% larger larvae than their Boat Ramp site counterparts (Figure 5; LM; *p* < .0001; Marina site 0.092 ± 0.001 mm^2^; Boat Ramp site 0.087 ± 0.001 mm^2^ [mean ± SE]). Additionally, there was a significant difference in size between larvae spawned before versus after copper exposure (LM; *p* = .002). Pairwise comparisons within each population showed that the Marina site mothers were driving this pattern and produced larger larvae in response to copper exposure whereas there was no significant difference in larval size before and after exposure in the Boat Ramp population (pairwise comparison; Marina site *p* = .002; Boat Ramp site *p* = .47).

## 4 DISCUSSION

We found different transgenerational responses to copper toxicity across our two populations. Maternal exposure to copper immediately prior to reproduction reduced offspring growth and survival in one population that lacked previous historical exposure, (Boat Ramp site) but not in another that had a documented exposure history (Marina site) (Figures 3 and 4). In individuals from the Marina site, neither maternal treatment nor offspring treatment affected subsequent offspring growth or survival with all individuals performing comparably across all treatments. However, we did find evidence that parental treatment increased subsequent larval size in this Marina population suggesting adaptive maternal priming or provisioning rather than no transgenerational effect. Overall, these results demonstrate variation among neighboring populations in their TGP response to exposure and these among‐population differences were consistent with our expectations. Although a greater number of populations with different exposure histories would be needed to be more certain, our data do support the hypothesis that evolutionary history with a selective agent influences the strength and direction of transgenerational effects.

The lack of any copper effect on growth and survival in the Marina population with greater historical exposure suggests that local adaptation may maintain fitness in the presence of this stressor. However, the increased size of larvae from this population indicates that maternal priming and/or provisioning may at least in part underlie this apparent adaptation. Mothers from the Marina site had larger larvae, in general, and created larger larvae in response to acute copper exposure. In contrast, mothers from the Boat Ramp site lacking this historical exposure produced smaller larvae and did not appear to increase larval size in response to acute copper exposure (i.e., in the Boat Ramp site, we did not find evidence for adaptive maternal priming; Figure 5). *Bugula neritina* larvae are lecithotrophic or non‐feeding and therefore all of the energy and nutrition to complete development are provided by their mothers. Increased larval size is directly correlated to larger energy reserves and has been linked to better survival and growth in adult colonies (Marshall et al., 2003). Previous work has also shown that *B. neritina* mothers are capable of controlling larval size and provisioning, with stressed mothers often producing larger larvae to compensate for the suboptimal environment (Marshall et al., 2006). Larval size at emergence is also predictive of time spent swimming (larger larvae have more resources that allow them to swim longer), which could also serve as a mechanism for larvae to escape stressful conditions (Marshall & Keough, 2003). This maternal plasticity may be an adaptive form of transgenerational plasticity to counter the negative carryover effects we see in more copper‐naïve populations and would have been missed by looking at the growth and survival data alone.

Acute exposure to copper in parent colonies from the Boat Ramp site produced a negative carryover effect (Uller et al., 2013; Uller & Pen, 2011) with offspring from parents exposed to copper having lower survival and growth rates. Though growth appears to be affected primarily by parental treatment regardless of offspring treatment, survival depended on both. Offspring from mothers exposed to copper that were exposed to copper themselves had the lowest survival rates overall, further suggesting a negative carryover effect. In this population that lacks an evolutionary history of copper exposure, both growth and survival of the offspring are negatively affected by maternal exposure to copper supporting a condition‐dependent maternal constraint effect on offspring.

Overall, we found differing transgenerational patterns across the two populations studied that may be linked to differential ability to produce larger larvae and manipulate larval size in response to stress. However, this conclusion should be tempered by the fact that we only examined two populations. Our results appear to align with different pieces of the maternal effects experiment run by Marshall (2008) with acutely copper‐exposed mothers producing larger larvae (as seen in our Marina population) but persistent negative carryover effects post‐metamorphosis on offspring from acutely copper‐exposed mothers (as in our Boat Ramp population), although it is unclear what the ambient copper level was in that study. This variation in responses across three different populations further supports the need for studies examining multiple populations with varying historical and environmental contexts. Theory predicts that a population with variable, but predictable stress should promote adaptive transgenerational plasticity (TGP) (Bonduriansky et al., 2012; Salinas et al., 2013). In this case, we know that the Marina site had higher copper concentrations, during our point survey but also on average and across time, than the Boat Ramp site (Mass spectrometry data, Blake et al., 2004; Schiff et al., 2007). But copper concentrations can still vary considerably over small spatial scales especially when its source is leaching from a stationary object or the source may leave for periods of time (such as boats) (Marshall & Keough, 2003; Miller, 1946). At the Boat Ramp site, we hypothesize that the low levels of copper exposure over time were insufficient to select for adaptation to acute copper exposure, leading to a detrimental or negative carryover effect when such exposures occurred in our experiment.

Further experiments including a larger number of populations with known long‐term copper exposure history would advance our understanding, but such histories are rarely known. Indeed, we specifically selected these sites due to their known history and proximity of just a few kilometers, which helped limit (but did not eliminate) confounding factors. Choosing sites along stress gradients with known patterns of variance (temporal or spatial) or predictability (autocorrelation) would be particularly fruitful and would allow for theoretical predictions to be tested as evolutionary histories are challenging to manipulate outside of theoretical/modeling space or with non‐model or longer‐lived species (Burgess & Marshall, 2014; Colicchio & Herman, 2020; Yin et al., 2019). However, such a gradient must be over sufficient spatial scale to limit migration among sites, but not such great distances to introduce confounding environmental variables. Other future studies exploring multi‐generational effects (e.g., grandparental effects; Bell & Hellmann, 2019) as well as examining whether the ontogenetic timing of exposure has an effect, particularly in this biphasic organism with dramatically different selection pressures during the larval versus post‐metamorphic adult phases would be logical next steps (Donelan et al., 2020; Donelson et al., 2018; Sobral et al., 2021).

These plasticity patterns may also contribute to our understanding of species' ability to rapidly adapt to human‐induced ecological changes as well as species invasion patterns. *Bugula neritina* is a successful invasive species found worldwide that is dealing particularly well with coastal development and heavy metal pollution from human activity (Mackie et al., 2006). Previous literature has linked invasion success in both terrestrial and marine environments with higher levels of plasticity (Davidson et al., 2011; Smith, 2009). While less is known specifically about the role of transgenerational plasticity in invasion, there is empirical evidence for its role in the success of invasive plants (Dyer et al., 2010; Fenesi et al., 2014) and theoretical work predicting its importance in human‐altered environments (Donelan et al., 2020). Studies comparing TGP patterns across native and invasive species complexes (such as *B*. neritina compared to its native congeners) could increase our understanding of the role of TGP in human‐altered environments and species invasions.

While TGP has proven to be a ubiquitous form of plasticity across a broad variety of taxa (Uller et al., 2013; Yin et al., 2019), explaining the broad variation in TGP observed among species and populations remains an important challenge. In light of increasing human disturbance in the natural environment, understanding how and when species are able to acclimatize or adapt quickly is an increasingly important area of research. We found two distinct patterns in response to multigenerational stress consistent with the idea that TGP is a locally adaptive response that may be selected for or against based on past exposure histories within a population. Future studies testing theoretical predictions with empirical data incorporating multiple populations along known environmental gradients and incorporating multiple metrics of performance and possible plasticity mechanisms should prove insightful.

## AUTHOR CONTRIBUTIONS

**Isabelle P. Neylan:** Conceptualization (lead); data curation (lead); formal analysis (lead); funding acquisition (lead); investigation (lead); methodology (lead); project administration (lead); resources (equal); visualization (lead); writing – original draft (lead); writing – review and editing (equal). **Andrew Sih:** Conceptualization (equal); funding acquisition (equal); resources (equal); validation (equal); writing – review and editing (equal). **John J. Stachowicz:** Conceptualization (equal); funding acquisition (equal); methodology (equal); resources (equal); supervision (equal); validation (equal); writing – review and editing (equal).

## CONFLICT OF INTEREST

The authors declare that they have no known competing financial interests or personal relationships that could have appeared to influence the work reported in this paper.

## Data Availability

The data have been deposited and are available through Figshare https://doi.org/10.6084/m9.figshare.19638783.v1 (Neylan, 2022).
